# A Cross‐Lagged Panel Analysis of Cortisol Levels and Internalizing Behaviors in Children Born Very Preterm Across Early Childhood: Associations Differ for Boys and Girls at Age 1.5 Years

**DOI:** 10.1002/dev.70064

**Published:** 2025-07-21

**Authors:** Mia A. McLean, Joanne Weinberg, Anne R. Synnes, Steven P. Miller, Ruth E. Grunau

**Affiliations:** ^1^ Department of Pediatrics University of British Columbia Vancouver British Columbia Canada; ^2^ BC Children's Hospital Research Institute Vancouver British Columbia Canada; ^3^ Department of Psychology and Neuroscience Auckland University of Technology Auckland New Zealand; ^4^ Department of Cellular and Physiological Sciences University of British Columbia Vancouver British Columbia Canada

**Keywords:** child sex, cortisol, HPA axis, internalizing behaviors, preterm

## Abstract

Children born very preterm (≤32 weeks’ gestation) are exposed to considerable stress in the neonatal period that, in turn, is associated long‐term with altered physiological stress reactivity and regulation, as well as increased internalizing (anxiety and depressive) behaviors. Whether cortisol levels are related to evolving internalizing behaviors in this population has not been evaluated to our knowledge. The present study investigated the association between cortisol reactivity to a cognitive assessment in a novel clinic environment and parent‐reported internalizing behaviors both concurrently and across ages in children born very preterm and examined whether relationships differed by biological sex at birth. Total cortisol output (AUCg) and reactivity (AUCi) were calculated from saliva assayed across age‐appropriate cognitive tasks, and parents reported on their child's behavior at ages 1.5, 3, and 4.5 years. Valid cortisol data at one or more assessment points were available from 174 participants. Random‐intercept cross‐lagged panel models showed no longitudinal relationships between internalizing behaviors and cortisol output (AUCg, AUCi). Follow‐up multilevel models revealed that the relationship between cortisol AUCg and internalizing behaviors was specific to girls at age 1.5 years. Findings highlight the importance of examining sex differences in biobehavioral relationships across development. Future research should consider factors that may attenuate these relationships across development.

## 1 Introduction

Infants born at extremely low gestational age (24–28 weeks’ gestation) and very low gestational age (29–32 weeks’ gestation), are born very preterm (24–32 weeks’ gestation), and represent a particularly vulnerable population. While survival of the tiniest babies has improved, neurodevelopmental outcomes persist (Younge et al. 2017). They are born during a period of rapid physiological and neurological immaturity (Ranger et al. 2022; Volpe 2009), including maturation of the hypothalamic–pituitary–adrenal (HPA) axis. These infants are exposed to adverse environmental exposures, including neonatal pain/stress and environmental disruptions, and are often separated from their parent(s) for long periods while hospitalized in the neonatal intensive care unit (NICU). Repetitive exposure to NICU stressors during fetal development ex utero is associated with altered early brain architecture (Boggini et al. 2021), which is related to early biological and behavioral functioning (Grunau et al. 2021; Lammertink et al. 2021).

In the immediate NICU period, these infants display dampened physiological stress responses (Grunau et al. 2005; Mörelius et al. 2016). This dampened response is thought to be a result of downregulation during a period of chronic stress exposure. In late infancy and toddlerhood, current evidence suggests children born very preterm display a switch to elevated cortisol response to stressors relative to those born full term (Brummelte, Grunau, Zaidman‐Zait, et al. 2011; Grunau et al. 2007; Stoye et al. 2022). At school age, both a dampened cortisol response to stress and cumulative cortisol levels (Brummelte et al. 2015; Grunau et al. 2013; Maurer et al. 2016) as well as hyperactivity (increased cortisol awakening response and responsivity to stress) have been reported (Quesada et al. 2014; Urfer et al. 2021). Sustained activation in toddlerhood may be related to early sensitization of the stress system to environmental influences (Boyce et al. 2021; Heim and Nemeroff 2001), including emerging social interactions, resulting in continued hyperactivity or downregulation in later childhood. By youth and adulthood, studies demonstrate blunted cortisol to stress (Kaseva et al. 2014) and less cortisol (lower morning cortisol and flatter diurnal curve) across the day (Lee et al. 2014) for those previously born preterm. Higher cortisol closer in time to acute stressors, then downregulation of cortisol long after chronic stress, is consistent with human studies on chronic stress (Duan et al. 2013; Yehuda 2001). Most recently, we showed that children born at extremely low gestational age (24–29 weeks’ gestation), relative to those born at very low gestational age (29–32 weeks’ gestation), show an elevated cortisol response upon arrival at neonatal follow‐up clinic, followed by a decrease across cognitive assessment at ages 1.5, 3, and 4.5 years, coupled with an overall decrease in average cortisol levels across ages (McLean et al. 2024).

For decades, developmental scientists have examined atypical physiological stress reactivity as a correlate of early adversity and as a contributor to social and emotional difficulties in children and adolescents (Lupien et al. 2009). However, research examining these relationships in children born very preterm is scant. This is surprising as children born very preterm display elevated risk of internalizing problems in comparison to children born full term (Gerstein et al. 2017) as early as toddlerhood (Vinall et al. 2013). Of relevance to the current study, we have recently shown that greater neonatal pain‐related stress exposure during the NICU stay is associated with elevated internalizing behaviors across ages 1.5, 3, 4.5, and 8 years (McLean et al. 2022). Moreover, we have demonstrated a relationship between elevated cortisol levels across cognitive assessment and concurrent behaviors specific to internalizing (anxiety, withdrawal) at age 1.5 years in two independent cohorts of children born very preterm (Brummelte, Grunau, Zaidman‐Zait, et al. 2011; McLean et al. 2023). Interestingly, in a previous cohort, we found that the relationship was no longer evident at age 8 years (Brummelte et al. 2015). Whether increases in stress responses, as indexed by cortisol levels, are related to behavioral displays of internalizing behaviors in early childhood in this population is unknown.

Given that emotion regulation and stress are tightly coupled across development (Krkovic et al. 2018; Wang and Saudino 2011), and difficulties with emotion regulation may increase vulnerability for internalizing behaviors (Hostinar and Cicchetti 2020), in the current study, we investigate how persistent alterations in HPA axis activity relate to internalizing behaviors across early childhood. A large body of work demonstrates that early adversity in the form of deprivation, for example, previously institutionalized children, is related to sustained hypocortisolism (lower basal levels, a flatter diurnal pattern, and blunted stress responses; Heim et al. 2000) across early childhood (Koss and Gunnar 2018) and to behavior problems. However, the timing of stress exposure for children born very preterm, as it coincides with the development of the HPA axis, overlaps in part with that of children exposed to in utero stress exposure. Specifically, longitudinal studies suggest that exposure to maternal stress in utero is related to hyperactivity of the HPA axis, including elevated stress response and a greater cortisol awakening response, in early childhood (Gunnar and Howland 2022; McLean, Simcock, et al. 2020; Yong Ping et al. 2015) and precedes the development of anxiety symptoms (McLean, Simcock, et al. 2020). Prior research investigating longitudinal biobehavioral associations in normative populations (Laurent et al. 2015) and early adversity cohorts (Koss et al. 2016; McLean, Simcock, et al. 2020) has not typically accounted for early internalizing behaviors, that is, behavior problems prior to assessment of cortisol, limiting our understanding of the likely bidirectional relationship between behavior and stress physiology across time (Perry et al. 2020). Moreover, the effects of acute changes in stress‐related hormones on behavior may be distinct from the long‐term effects of chronic elevations in cortisol. Yet, to date, no study has examined the longitudinal relationship between cortisol and internalizing behaviors in children born very preterm (to our knowledge).

By examining how internalizing behaviors, commonly displayed in this population, are related to later physiological stress regulation, we may come to understand how children born very preterm with elevated internalizing behaviors respond to environmental stressors beyond NICU stay. Children born very preterm are faced with ongoing external environmental pressures related to their difficulties in executive functions and social communication skills. Related work in children born very preterm has found alterations in key limbic brain regions (Brummelte et al. 2015; Duerden et al. 2016) and networks (Lammertink et al. 2022) involved in HPA axis functioning, evident in early life and persisting through school age. Importantly, changes to brain architecture in such regions are related to concurrent internalizing behaviors (Chau et al. 2019; Gilchrist et al. 2023, 2022). As internalizing behaviors typically increase with age in this population (McLean et al. 2022; Scott et al. 2018; Yates et al. 2020), in line with research in normative populations characterizing internalizing behaviors as indicative of greater behavioral expression of negative emotions (De Pauw and Mervielde 2010) and threat‐related attention bias (Abend et al. 2020; Fu and Pérez‐Edgar 2019; McLean, Van den Bergh, et al. 2020), we hypothesize that children exhibiting greater internalizing behaviors will display heightened responsivity to future stressors. Therefore, in the current study, we examined relationships between behavior and HPA axis functioning both concurrently and across age.

Another factor that is rarely investigated in children born very preterm is the existence of sex differences in physiological stress system functioning in relation to behavior. Empirical investigations of sex differences in physiological stress system functioning in early childhood (Cowell et al. 2021; Hodes and Epperson 2019) and neuroscience and psychiatry research more broadly (Rechlin et al. 2022; Stroud et al. 2011) are rare. In animal models, sex differences are evident in the neonatal period, with females demonstrating a more robust response to acute stress as indexed by increased cortisol (Heck and Handa 2019; Panagiotakopoulos and Neigh 2014). Sex differences in HPA axis, as well as regulatory limbic structures and expression of HPA‐related genes, may underpin these differences (Heck and Handa 2019). Of the handful of studies that have examined sex differences in HPA axis functioning in the preterm population, findings are mixed (Brummelte et al. 2015; Grunau et al. 2013; McLean et al. 2021; Quesada et al. 2014; Stoye et al. 2022). Inferential understanding is hindered by several factors that could influence findings, such as variation in the type of stressor, index of HPA axis functioning (diurnal pattern or stress reactivity), and child age at assessment. Moreover, few examine whether sex‐specific alterations are related to neurodevelopmental outcomes, and thus, the implications of sex‐specific stress system functioning in this population are not understood. Of most relevance to the current work, in our prior work in this cohort, we found biobehavioral relationships differed by sex, such that greater sensory processing problems were associated with higher average cortisol output across assessment for girls but not for boys at 4.5 years (McLean et al. 2021). The dearth of research investigating sex‐specific biobehavioral relationships considering stress physiology is problematic, given the well‐established sex differences related to prematurity (Peacock et al. 2012), with males at greater risk of neonatal outcomes, including death and oxygen dependency, as well as disability and cognitive delay at follow‐up. In contrast, females are at greater risk of internalizing behaviors in later childhood through adolescence in the general population (Altemus et al. 2014).

In the current study, we examined the relationships between behavior and HPA axis functioning both concurrently and across age. We also investigated sex differences in the association between physiological stress regulation and internalizing behaviors, a key difficulty for children born very preterm, at ages 1.5, 3, and 4.5 years. Our repeated‐measures design within a cohort of children born very preterm is internationally unique and has the potential to inform understanding of the developmental pathways of vulnerability of the HPA axis, as well as the interplay between physiological stress regulation and behavior across early childhood in this population. Given the scarcity of work examining HPA axis functioning in this population across early childhood, findings from our current study will be important in identifying whether early perturbations persist and alter later behavioral functioning. Such work will add weight to accumulating evidence in humans that the prenatal period is a sensitive period of development (Gabard‐Durnam and McLaughlin 2020; Gunnar and Howland 2022).

In our work, due to ethical concerns around stressing vulnerable preterm children, we examine patterns and levels of cortisol across age‐appropriate developmental testing. Administered in a clinic environment, both the unfamiliar setting and the cognitive tasks are challenging for preterm children (Grunau et al. 2004). Herein, we refer to developmental testing as the “cognitive assessment.” Across two independent prospective longitudinal cohorts, our group has shown that children born very preterm respond to this assessment with elevated cortisol output prior to the cognitive assessment (pretest), followed by a decrease throughout the assessment to the end, at ages 1.5, 3, 4.5, and 8 years (Brummelte, Grunau, Zaidman‐Zait, et al. 2011; Brummelte et al. 2015; McLean et al. 2021). Here, we build on our prior findings to examine concurrent and long‐term relationships between internalizing behaviors and cortisol output in response to cognitive assessment across ages 1.5, 3, and 4.5 years. Second, we considered whether relationships differed for boys and girls.

## 1 Methods

### 1.1 Participants and Procedures

Infants born very preterm (24–32 weeks’ gestational age) recruited between 2006 and 2013 from the Level III Neonatal Intensive Care Unit (NICU) at BC Women Hospital in Vancouver Canada returned for follow‐up at corrected ages 1.5, 3, and 4.5 years as part of a prospective longitudinal study of very preterm children. Follow‐up at 4.5 years was completed during the year 2018. Neurodevelopmental assessment at each age was carried out by experienced staff (physiotherapist, psychologist, or developmental consultant) in the Neonatal Follow‐up Program at the Children's & Women's Health Centre of British Columbia. Child neurodevelopmental assessment, the cognitive assessment, was the Bayley Scales of Infant Development 3rd Edition (Bayley‐III; Bayley 2005) at ages 1.5 and 3 years and the Wechsler Preschool and Primary Scale of Intelligence‐ IV (WPPSI‐IV) at age 4.5 years. Saliva samples were collected at three time points across assessment: first at *Pretest* (∼15 min after the child was settled following arrival at the center), then after the cognitive assessment (reflecting cortisol levels *During* cognitive assessment), and finally at the *End* of the study visit (∼20 min after the cognitive assessment). All assessments were carried out in the morning; see Table S1 for collection times across ages.

After exclusions, 174 children who attended developmental assessment and had valid cortisol data at all three collection time points (Pretest, During, End) at ages 1.5 years (*n* = 102), 3 years (*n* = 127), or 4.5 years (*n* = 121) were included in the current study. Exclusions included major sensorineural hearing impairment (1), cognitive impairment at age 4.5 years (WPPSI‐IV IQ <70) (8), major genetic syndrome (2), nonambulatory cerebral palsy (2), and severe autism (1). Cortisol data were excluded if the child was on medication that affects cortisol levels (10 at 1.5 years, two at 3 years, six at 4.5 years) and when the child had food or drink within 20 min prior to any saliva collection (25 at 1.5 years, 15 at 3 years, 11 at 4.5 years).

The study was approved by the University of British Columbia Clinical Research and BC Women's Hospital Ethics Board (H05‐70579) and was conducted in accordance with the Declaration of Helsinki (1964). Parent consent was obtained at recruitment and again at each follow‐up visit. Additional ethics applications were submitted for follow‐up study and renewed every year to ensure our studies cover current research questions and are conducted according to the university's ethics standards.

### 1.2 Measures

#### 2.2.1 Child Cortisol

As described in procedures, saliva samples were collected at three time points across the study visit (Pretest, During, and End) using Salivettes (Salimetrics; Salimetrics LLC, State College, PA). Saliva samples were stored at −20°C for less than a year after collection and assayed in batches of 48 children per run using the Salimetrics High Sensitivity Salivary Cortisol Enzyme Immunoassay Kit for quantitative determination of salivary cortisol. All samples were assayed in duplicate. The intra‐ and interassay coefficients of variation were 5.04 and 6.58, respectively.

Given that cortisol levels peak 15–20 min post‐stressor/challenge, the first saliva sample is an indicator of “Pretest” or cortisol levels upon arrival at the testing center, the second saliva sample indicates cortisol levels “During” the cognitive assessment, and the third sample indexes cortisol at the “End” of the cognitive assessment. We examined cortisol output across assessment using two indices: area under the curve with respect to ground (AUCg) and area under the curve with respect to change over time (AUCi). AUCg is used to derive an estimate of total cortisol output, capturing both intensity and sensitivity. AUCi is used to derive a cortisol response over an event period. AUCi measures change in cortisol over repeated samples, independent of prechallenge cortisol concentrations, and can be thought of as an index of sensitivity of the system in response to an event (Fekedulegn et al. 2007). Formulae for AUCg and AUCi are derived from the trapezoid formula (Pruessner et al. 2003). AUCg is the AUC across samples, and AUCi equates to AUCg minus the AUC below baseline (here, Pretest). Higher scores on AUCg represented greater cortisol output across cognitive assessment, and higher scores (less negative) on AUCi represent less decrease in cortisol from Pretest.

#### 2.2.2 Child Internalizing Behaviors

At each age, parents reported on their child's behaviors using the Child Behavior Checklist 1.5–5 years (Achenbach and Rescorla 2000). The CBCL comprises 101 items; parents report the frequency of child behaviors (0 = *not true*, 1 = *somewhat or sometimes true*, 2 = *very true or often true*). The CBCL has high internal consistency (Cronbach's α’s range from 0.78 to 0.97) and has been used with preterm children (e.g., Gerstein et al. 2017; Gray et al. 2004). In this study, we used the Internalizing Behavior (anxious/depressed, emotionally reactive, withdrawal behaviors, somatic complaints) subscale *T*‐score (standardized for child age and sex).

### 1.3 Statistical Analysis

Analyses were conducted in R (version 4.1.6, R Core Team 2021). Given a nonnormal distribution, AUCg was log transformed at each age, and both AUCi and AUCg variables were winsorized for outliers (Miller and Plessow 2013). First, we examined descriptive statistics, correlations among study variables, and consistency of the CBCL Internalizing scores across ages 1.5, 3, and 4.5 years, using Cronbach's alpha. Then we conducted random‐intercept cross‐lagged panel models (RI‐CLPMs) to examine cross‐sectional and longitudinal relationships between internalizing behaviors, AUCg and AUCi for all children across ages 1.5, 3, and 4.5 years, accounting for within‐participant variation in intercept (age 1.5 years). While the traditional CLPM fails to separate between‐person differences from within‐person differences over time, the inclusion of a latent random intercept in RI‐CLPMs affords this separation (Mulder and Hamaker 2021). While Autoregressive Latent Trajectory models with standardized residuals extend upon RI‐CLPM methods to consider between‐person differences in within‐person development via latent slope estimation, such analysis requires four or more time points of data (Mund and Nestler 2019). We highlight this difference for the reader, as this is an important distinction. Analyses used herein do not provide information regarding developmental growth/change over time in variables of interest (internalizing behaviors, AUCi, AUCg). Rather, RI‐CLPMs describe correlations between trait‐like between‐person differences (random intercepts) in variables in addition to autoregressive (concurrent) and cross‐lag relationships for within‐person differences. For example, a positive autoregressive parameter would indicate that when a child's AUCi is above his or her average level (person‐specific mean) of AUCi, this child is expected to score above his or her person‐specific mean in AUCi at the subsequent time point. See Mulder and Hamaker (2021) and Mund and Nestler (2019) for a detailed explanation of RI‐CLPMs and further discussion of modeling techniques.

Data were analyzed using the “lavaan,” “lmerTest,” and “lme4” packages in R. Missing data were handled using full‐information maximum likelihood. Separate models were run for cortisol AUCi and AUCg in relation to internalizing behaviors across ages. We examined sex differences in path analyses by running a multigroup RI‐CLPM. These models were compared with traditional CLPMs to test whether stable between‐person differences exist and with models without concurrent and cross‐lagged effects to test the necessity of cross‐lagged paths. Model fit was determined by examining global and local fit indices (Kline 2023) and conducting chi‐square difference tests when comparing nested models. To further explore cross‐lag analyses by child sex, multilevel models were tested.

## 2 Results

### 2.1 Description of the Cohort

Cohort descriptives are summarized in Table 1. Of the 174 children included in the current study, 93 (53%) were male at birth and 111 (64%) were born at extremely low gestational age (24–28 weeks). Mothers of children included in the study were well educated (85% graduate or postgraduate level). Children were born on average at 28 weeks’ gestation (*SD* = 2.3). Cronbach's alpha on CBCL Internalizing T‐scores across ages 1.5, 3, and 4.5 years (α = 0.751) indicated that the CBCL Internalizing score provided a consistent construct from toddlerhood through early childhood in this sample of children born very preterm. CBCL Internalizing scores did not differ by child sex at any age (see Table 1).

**TABLE 1:** Descriptive statistics for demographic and neonatal clinical factors.

	Mean (*SD*)		
	Range (min–max)		
	Boys(*N* = 93)	Girls(*N* = 81)	*p*‐value
Neonatal clinical factors			
Gestational age at birth (weeks)	28.08 (2.31)	27.97 (2.31)	0.756
	24.29–32.00	24–32.29	
Gestational age group, *n* (%)			
Extremely low gestational age (24–28 weeks)	57 (61)	54 (67)	0.462
Very low gestational age (29–32 weeks)	36 (39)	27 (33)	
Illness severity at birth^a^	14.23 (13.55)	10.99 (11.46)	0.096
	0–57	0–56	
Days on respiratory support	46.41 (36.05)	48.86 (37.06)	0.661
	0–108	0–109	
Surgery, 1+	27 (29)	25 (31)	0.628
Culture positive infection, 1+	46 (49)	28 (48)	0.859
Number of painful procedures	116.17 (77.98)	117.23 (72.46)	0.927
	27–385	14–323	
Demographics			
Maternal age at birth (years)	32.7 (5.1)	32.5 (5.5)	0.815
	22.1–45.9	21.8–44.3	
Maternal level of education, *n* (%)			0.800
Primary or secondary school graduation	11 (12%)	11 (14%)	
Partial or complete undergraduate degree	65 (71%)	52 (67%)	
Postgraduate university degree	15 (16%)	15 (19%)	
Maternal ethnicity, *n* (%)			1.00
White Caucasian	56 (62)	48 (62)	
Other	35 (38)	53 (68)	
Paternal age at birth (years)	35.3 (5.7)	34.2 (6.0)	0.245
	20.9–50.7	22.0–48.9	
Paternal level of education, *n* (%)			0.584
Primary or secondary school graduation	17 (19%)	20 (26%)	
Partial or complete undergraduate degree	59 (67%)	48 (62%)	
Postgraduate university degree	12 (14%)	9 (12%)	
Paternal ethnicity, *n* (%)			0.117
White Caucasian	70 (79%)	53 (68%)	
Other	19 (21)	25 (32)	
CBCL Internalizing Behavior *T*‐score			
Age 1.5 years	43.7 (9.0)	45.2 (9.8)	0.346
	29.0–66.0	29.0–83.0	
Missing	17	12	
Age 3 years	47.8 (10.3)	48.0 (9.7)	0.930
	29.0–80.0	29.0–78.0	
Missing	4	10	
Age 4.5 years	47.7 (11.6)	49.8 (12.3)	0.281
	29.0–79.0	29.0–77.0	
Missing	8	12	

^a^ Neonatal Acute Physiology (SNAP)‐II score.

**FIGURE 1:**
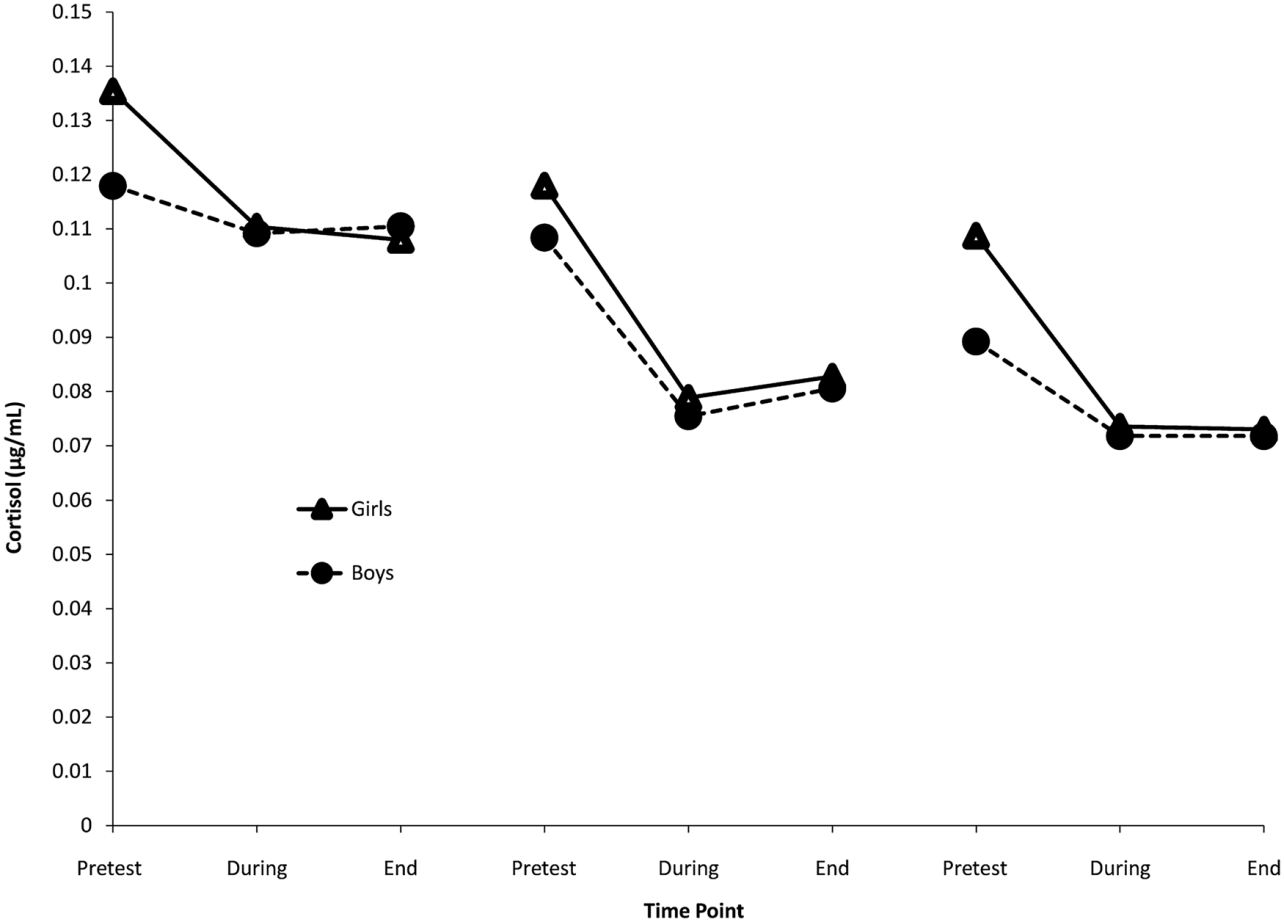
Pattern of cortisol output in response to cognitive challenge across 1.5, 3, and 4.5 years in girls and boys born very preterm. Cortisol values are back‐transformed.

Figure 1 displays the pattern of cortisol output across ages 1.5, 3, and 4.5 years for boys and girls. At each age and for both sexes, cortisol levels were highest prior to cognitive assessment, followed by a decrease during testing and remaining stable through the end of the assessment.

Pearson correlations between study variables for boys and girls at each age are presented in Table 2. Greater internalizing behaviors were related to higher AUCg for girls but not for boys at age 1.5 years. Cortisol levels were not related to the time of cortisol collection.

**TABLE 2:** Correlations between study variables for boys and girls.

			Girls								
			1	2	3	4	5	6	7	8	9
Boys	1	1.5‐year AUCg		0.08	0.14	−0.25	−0.16	0.23	0.28^^^	0.08	0.40^**^
	2	3‐year AUCg	0.1		0.38^**^	0.11	−0.39^**^	−0.29	−0.29^*^	0.03	−0.05
	3	4.5‐year AUCg	0.04	0.25		−0.31	−0.17	−0.14	−0.09	0.03	0.07
	4	1.5‐year AUCi	−0.53^****^	−0.12	−0.05		−0.07	−0.17	−0.21	−0.12	−0.19
	5	3‐year AUCi	−0.01	−0.37^**^	−0.23	−0.02		0.35^*^	−0.1	−0.18	0
	6	4.5‐year AUCi	0.02	−0.24	−0.15	0.07	0.1		−0.11	−0.21	0.01
	7	1.5‐year Internalizing	−0.16	0.08	0.23^*^	−0.03	−0.26^*^	−0.05		0.48^****^	0.44^***^
	8	3‐year Internalizing	−0.09	0.1	0.05	−0.15	−0.15	0.03	0.58^****^		0.53^****^
	9	4.5‐year Internalizing	−0.06	0.13	0.03	−0.01	−0.13	0.02	0.40^***^	0.62^****^	

^*p* < 0.01; *p < 0.05; **p<0.01; ***p < 0.001; ****p < 0.0001.

### 2.2 RI‐CLPM AUCi, AUCg, and Parent‐Reported Internalizing Behaviors

Complete reporting of cross‐lagged analyses is available in the Supporting Information Results. Random‐intercept cross‐lagged path analyses showed no concurrent or cross‐lagged relationships between internalizing behaviors and AUCg or AUCi across ages (see Supporting Information Results and Figure S1).

The same was true for RI‐CLPMs examining sex differences: no concurrent or cross‐lagged relationships were evident (see Supporting Information Results and Figure S2). There was a trend for girls with higher CBCL Internalizing (between‐individual) to display higher than average (within‐individual) Cortisol AUCg at 1.5 years (Figure S2).

As RI‐CLPMs did not indicate significant cross‐lagged or concurrent associations, we followed up our investigation of the association between CBCL Internalizing and Cortisol AUCg using a multilevel modeling approach, which allows for estimation of individual differences in overall levels (random intercepts) and potential changes over time in a more parsimonious manner. MLM allows for estimation of overall differences across time while accounting for individual differences (random intercepts) and is more reliable in estimating change across time than latent growth curve models when only three time points are included. This is important given the nonlinear change in Cortisol AUCg in our current data. The two‐way interaction of Child Age × Internalizing was not significant, *F*(2) = 1.30, *p* = 0.273. The three‐way interaction of Child Age × Child Sex × Internalizing was significant (see Tables S2 and S3 and Figure S2). Above and beyond gestational age at birth, girls whose parent reported higher Internalizing at age 1.5 years displayed elevated AUCg across cognitive assessment at age 1.5 years (*B* = 0.023, *SE* = 0.009, *p* = 0.010). However, this relationship was not evident at age 3 years (*B* = 0.00, *SE* = 0.01, *p* = 0.910) or 4.5 years (*B* = 0.00, *SE* = 0.01, *p* = 0.521). For boys, AUCg was unrelated to Internalizing at ages 1.5 years (*B* = −0.01, *SE* = 0.01, *p* = 0.091), 3 years (*B* = 0.00, *SE* = 0.01, *p* = 0.565), and 4.5 years (*B* = −0.00, *SE* = 0.01, *p* = 0.743). Fixed effects accounted for 10.2% of the variance in outcome, and 16.1% of the variance was accounted for by individual variation in the intercept (Cortisol AUCg at age 1.5 years).

**FIGURE 2:**
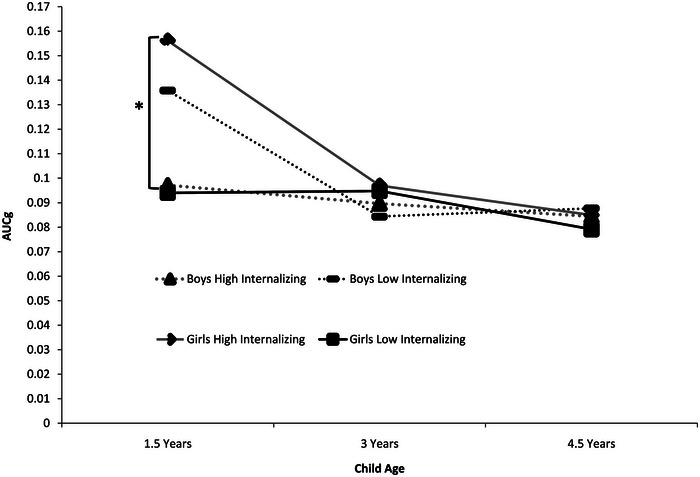
Cortisol AUCg in relation to CBCL Internalizing Behaviors across 1.5, 3, and 4.5 years in boys and girls. Low Internalizing = *T*‐score of 37; High Internalizing = *T*‐score of 54. AUCg accounts for time across assessment and is back‐transformed.

The three‐way interaction of Child Age × Child Sex × Internalizing in relation to AUCi was not significant (see Tables S3 and S4).

## 3 Discussion

In the current study, we built on our prior work to examine the relationships between trajectories of cortisol output in response to cognitive assessment and internalizing behaviors concurrently and across early childhood in a current cohort. At 1.5 years, for girls only, greater concurrent average cortisol input Internalizing behaviors were associated with greater average cortisol output (AUCg) across assessment. No relationships were evident at ages 3 or 4.5 years in boys or girls. This is the first study, to our knowledge, to examine longitudinal relationships between internalizing behaviors and HPA axis functioning in children born very preterm.

In line with our prior work across two independent cohorts (Brummelte, Grunau, Zaidman‐Zait, et al. 2011; McLean et al. 2021, 2023), child cortisol levels were highest prior to cognitive assessment, followed by a decrease during testing and remaining stable through the end of the assessment. Importantly, Watterberg et al. (2019) reported a similar pattern of cortisol output across cognitive assessment in children born very preterm at age 6 years. In early childhood, children born full term display a similar pattern but lower cortisol levels prior to assessment and less cortisol output across cognitive testing (McLean et al. 2023). A comprehensive description and discussion of this pattern of cortisol output across ages have been reported previously (McLean et al. 2023, 2024). In brief, elevated cortisol levels prior to assessment may be indicative of anticipatory stress as children find the developmental assessment and the unfamiliar assessment challenging (Grunau et al. 2004). It is also possible that it reflects stressors immediately prior to arrival. A decrease across neurodevelopmental assessment may be indicative of increased engagement with the cognitive assessment or a return to baseline.

Contrary to our hypothesis, we did not find bidirectional relationships between cortisol levels and internalizing behaviors across child assessment ages. Although a significant amount of empirical work investigates the association between HPA functioning and concurrent or subsequent internalizing behaviors, relatively little work has been conducted in children born very preterm. Moreover, our work is unique in that we were able to consider both concurrent and cross‐lagged effects of biobehavioral associations. Our findings demonstrate the importance of investigating bidirectional effects of biobehavioral associations to better understand how HPA axis activity and internalizing behaviors are associated over time. Despite considerable between‐subject variation in cortisol levels and increased internalizing behaviors across age in this cohort (McLean et al. 2022), there may be too little within‐individual variability in internalizing symptoms and HPA axis activity over time to detect significant associations. However, we did find evidence of a relationship between heightened cortisol output and elevated internalizing behaviors at 1.5 years for girls but not for boys. This finding extends the limited prior work demonstrating a relationship between HPA axis activity and internalizing behaviors in children born very preterm to suggest that this relationship may be sex specific. As posited by the adaptive calibration model, sex differences may emerge in the context of environmental stressors, with females displaying greater stress reactivity related to withdrawn and vigilant tendencies (Del Giudice et al. 2011). For girls, greater withdrawal behaviors and fearfulness, key behaviors of internalizing behaviors, may predict a stronger biological response to novel testing environments in early infancy.

The onset of anxiety and fear‐related disorders peaks at 15 years of age (Solmi et al. 2022), increasing from childhood in a sex‐specific fashion, with females displaying greater internalizing behaviors across adolescence (Nivard et al. 2017; Zahn‐Waxler et al. 2008). In addition, the hypothalamic–pituitary–gonadal and HPA axes are tightly linked, with HPA axis regulation influenced by hypothalamic–pituitary–gonadal functioning (Da Silva 1999). Therefore, it would be informative for future work in this population to examine sex‐specific profiles of HPA axis regulation and biobehavioral relationships across schooling through adolescence.

It is also possible that increased cortisol levels prime the individual to experience increased fearfulness and withdrawal behaviors across a variety of challenging situations. At age 4.5 years in the current cohort, elevated average cortisol output was related to more sensory processing problems in very preterm girls but not boys (McLean et al. 2021). Both hypo‐ and hypersensitivity to sensory stimuli contribute to socialization challenges related to internalizing behaviors (Carpenter et al. 2019). While interrelated, empirical research suggests sensory processing difficulties typically precede the development of internalizing behaviors (Green and Ben‐Sasson 2010; Méndez Leal et al. 2023). Such findings are perhaps in contrast to our findings herein, suggesting differential rather than overlapping pathways when considering biobehavioral relationships following preterm birth. Still, we did not assess sensory processing behaviors during toddlerhood, and therefore, we cannot assess the specificity of this relationship across developmental stages. Together, our findings suggest that cortisol output in response to a challenging cognitive assessment in a novel, unfamiliar testing environment may be a physiological marker of behavior problems, particularly for girls born very preterm.

Our finding of a lack of association between internalizing behaviors and cortisol output during cognitive assessment across childhood is potentially mediated by parent mood and behavior not investigated in the current study. The HPA axis is regulated by parental behavior in young children (Gunnar and Hostinar 2015), and parenting support has been shown to ameliorate the long‐term effects of prematurity on the development of internalizing behaviors (Church et al. 2020; McLean et al. 2022; Treyvaud et al. 2009, Treyvaud et al. 2016; Vinall et al. 2013). In line with our understanding of sensitive periods of development (Boyce et al. 2021), girls born very preterm may be both more susceptible to and benefit most from parenting in the first years of life, at least in the context of a novel environment and cognitive assessment. In an earlier cohort at age 8 years, we showed that cortisol output in response to cognitive assessment was related to concurrent parent mood for girls, while boys’ cortisol output was related to degree of prematurity and associated NICU exposures and clinical factors (Brummelte et al. 2015; Grunau et al. 2013). In the current cohort, at age 3 but not 1.5 years, parent behaviors buffered long‐term effects of neonatal pain–stress and brain dysmaturation on internalizing behaviors, cognition, and motor behaviors; however, sex differences were not explored (McLean et al. 2022b; Miller et al. 2022). More research is needed to understand individual differences in developmental influences of parent attributes and mood, as well as child top‐down regulatory abilities that may help understand whether toddlerhood is a particularly sensitive period resulting in a window of opportunity following preterm birth.

The relationship between HPA axis function and internalizing behaviors was specific to AUCg, that is, indicative of total cortisol output and not change in cortisol across time, considering individual baseline levels (AUCi; Fekedulegn et al. 2007; Khoury et al. 2015). Studies that examine cortisol output in response to psychosocial stressors (e.g., Trier Social Stress Test) often report on AUCi only (Perry et al. 2020). However, disparate findings are evident in the literature. Of relevance, Blankenship et al. (2019) found that AUCg and not AUCi in response to a challenging puzzle task at preschool age was related to later hippocampal–midcingulate cortex connectivity 3 years later. Given the interrelationship between early life stress, hippocampal structure/function, HPA axis functioning, and internalizing behaviors, it is interesting to consider whether, at least in early childhood, total cortisol output is a more impactful measure of HPA axis negative feedback mechanisms of the hippocampus and therefore behavior in children born very preterm. In addition, prior work by our team has shown that clinical factors related to prematurity are associated with poorer hippocampal maturation across the neonatal period (Duerden et al. 2016). Total cortisol output may better capture baseline “trait‐like” cortisol output in addition to responsivity to context. Girls who typically display more internalizing behaviors are likely to show higher baseline cortisol and to be primed for a heightened response to a novel, potentially threatening environment and a subsequent challenging task. Alternatively, or in addition, a greater decrease in cortisol across assessment may suggest that girls are able to focus attention and process information to a greater extent across developmental assessment. Support for this hypothesis is clear when one considers the well‐established male vulnerability in premature‐born infants (Kozhemiako et al. 2020; Schindler et al. 2017). Still, whether these statistically independent constructs (Khoury et al. 2015) are related to physiologically distinct processes is, to the best of our knowledge, unknown. We are therefore cautious in our interpretation of an age‐dependent relationship between global HPA axis dysfunction and internalizing behaviors in girls born very preterm. Rather, our findings suggest that maternal‐reported internalizing behaviors in early toddlerhood are related to total cortisol output, and not change in cortisol levels, across cognitive assessment.

Our finding of cortisol hyperreactivity as related to greater internalizing in early childhood is in line with research examining biobehavioral outcomes associated with maternal stress in pregnancy. Interestingly, findings contrast with studies of children exposed to early adversity in infancy. A large body of work has demonstrated that previously institutionalized children show hyporeactivity of the HPA axis to stress across childhood (Gunnar and Howland 2022), suggesting that the timing of disruption to the HPA axis is important for understanding the nature of developmental patterns of altered HPA axis functioning in relation to a variety of emotional outcomes in at‐risk populations. While not studied here, we and others have previously found lower cumulative cortisol levels and a blunted cortisol response to stress in children born very preterm at school age (Brummelte et al. 2015; Grunau et al.^2013^) through to adulthood (Kaseva et al. 2014; Lee et al. 2014). Such a developmental pattern is in line with theories of allostatic load (McEwen 2002) and has been found in children exposed to poverty (Blair et al. 2011). Furthermore, outcomes and underlying biological mechanisms related to early adversity have been posited to differ, depending on domains of adversity exposure (threat, neglect; McLaughlin et al. 2020; McLaughlin and Sheridan 2016). Unlike children who are reared in orphanages in infancy, children born very preterm are exposed to repetitive daily threats from invasive procedures, as well as neglect in the form of parent separation. Future research could examine the relative contributions of NICU threat and deprivation to HPA axis dysregulation in children born very preterm. Recently, Stroud et al. (2024) demonstrated that exposure to early‐life threat, but not deprivation, was related to lower latent trait cortisol profiles across assessment waves spanning 3 months in adulthood. Preclinical studies are needed to further elucidate biological mechanisms.

The current cohort comprises more infants born at extremely low gestational age (24–28 weeks’ gestation) than infants born at very low gestational age (29–32 weeks’ gestation). Infants born at extremely low gestational age (extremely preterm) are at greater risk of poorer outcomes, and our prior work has demonstrated that those born extremely preterm display dysregulated physiological stress relative to those born later (Brummelte, Grunau, Zaidman‐Zait, et al. 2011; Grunau et al. 2007; McLean et al. 2021a, 2023). While a potential limitation, similar numbers of boys and girls were born extremely preterm. Moreover, we controlled for gestational age group across analyses, suggesting findings were specific to child sex. The lack of a full‐term control group is a limitation of the current study, as it hinders an understanding of normative responses to the cognitive assessment and thus whether relationships with internalizing established in the current work are adaptive or not. Still, we have previously demonstrated that full‐term children demonstrate lower cortisol levels, unrelated to internalizing behaviors, at 1.5 years (Brummelte, Grunau, Zaidman‐Zait, et al. 2011; McLean et al. 2023). Moreover, we found this pattern of cortisol output across developmental assessment across ages in two independent cohorts (Brummelte et al. 2015; Brummelte, Grunau, Synnes, et al. 2011; Grunau et al. 2007; McLean et al. 2021). Cortisol levels were measured across the cognitive assessment due to ethical reasons. Broader research has reported that eliciting a cortisol response to stress paradigms in toddlerhood and early childhood is difficult (Gunnar et al. 2009). The need for standardized stressor paradigms to assess cortisol responses systematically across infancy is clear (Puhakka and Peltola 2020). Future research will benefit from the use of a full‐term control group and a standardized stressor. Saliva samples were collected across the morning when children attended neonatal follow‐up clinic visits. Time of day of cortisol collection was not associated with cortisol levels despite the sharper decline of the cortisol circadian curve toward midday (Miller et al. 2016). Finally, despite group averages indicating decreases in cortisol from Pretest to During assessment, variation in the pattern existed. In related work, we have examined the cortisol pattern across the three time points rather than summary measures (McLean et al. 2024). Such analysis may be more sensitive to variation in individual sensitivity of the system to context; however, considering repeated measures within time points and across time points within a cross‐lagged structural equation model is complex, and we did not have the sample size to run such analyses.

Strengths of the current study include the collection of repeated saliva samples and parent reports of behaviors across three ages in early childhood. These data are unique in a prospective longitudinal cohort of children born very preterm, to our knowledge. Our high participant retention enabled the analysis and detection of sex differences in HPA axis functioning. Another clear strength of the current work is that analyses accounted for medications and food/drink intake that may affect cortisol levels.

In the current study, we did not include observed child behavior during developmental assessment. In doing so, future research may better understand the bidirectional nature of HPA axis regulation and real‐time regulatory behaviors in children born very preterm, as well as the influence of the cognitive assessment on physiological stress and behavior across ages. We have previously shown that children born preterm display greater anxiety‐like behaviors across cognitive assessment at ages 3 (Grunau 2003) and 9 years (Whitfield et al. 1997). Moreover, Future work could examine whether concurrent and longitudinal relationships between cortisol output and internalizing behaviors are evident in those most “at risk,” for example, infants born very preterm with greater medical/biological risk or those exposed to greater neonatal adversity independent of, or interaction with, child sex. We and others have shown altered cortisol reactivity to stress for those exposed to greater neonatal pain–stress (greater number of painful procedures; Church et al. 2020; Montirosso et al. 2016; Provenzi et al. 2016). More broadly, children exposed to more clinical factors (McLean et al. 2022; Ranger et al. 2014; Vinall et al. 2013) and those with altered brain maturation (Miller et al. 2022) and greater medical/biological risk (e.g., Treyvaud et al.^2012^; Doyle et al.^2015^) associated with prematurity are more vulnerable to internalizing behaviors and neurodevelopmental difficulties. In the current study, boys and girls did not differ in clinical exposures or illness severity at birth as related to degree of prematurity. Importantly, group averages minimize individual variation in pattern, and future work could consider defining nonresponses, as well as anticipatory and reactive responses to this task, as has been done with regard to standardized psychosocial stressors later in development (e.g., Herbison et al. 2016).

This is the first study, to our knowledge, to describe trajectories of HPA axis activity across early childhood as they relate to internalizing behaviors in children born very preterm. Further research is needed to understand the sex‐specific nature of early HPA axis programming in this population, as well as factors that attenuate this relationship across childhood. Continued research in sex differences in biobehavioral relationships across childhood in this population is a clear direction for future research. Our work emphasizes the importance of understanding the nature of child physiological stress regulation and biobehavioral relationships, utilizing longitudinal research designs.

## Ethics Statement

The study was approved by the University of British Columbia Clinical Research and BC Women's Hospital Ethics Board (H05‐70579) and was conducted in accordance with the Declaration of Helsinki (1964). Parent consent was obtained at recruitment and again at each follow‐up visit. Ethics applications were renewed every year to ensure our studies cover current research questions and are conducted according to the university's ethics standards.

## Conflicts of Interest

The authors declare no conflicts of interest.

## Supporting information




**Supplementary Table 1:** Time of cortisol collection at each study visit
**Supplementary Results** Complete reporting of Random Intercept Cross‐lag analyses for Cortisol AUCg, Cortisol AUCi and Internalizing
**Supplementary Fig.1:** Models examining the Bidirectional associations between Cortisol AUCg and Internalizing. (A) Random Intercept CLPM, (B) Random Intercept CLPM model including RI for Internalizing only and (C) CLPM
**Supplementary Fig.2:** *Models examining Cortisol AUCg and Internalizing across ages for boys and girls. Bidirectional association Cortisol AUCg and Internalizing for boys and girls, (A) Random Intercept model including Between factor for Cortisol AUCg, (B) Cross‐lag Panel model*
**Supplementary Table 2:** *Analysis of Variance Table for multilevel model examining relationships among Child age, CBCL Internalizing and Child sex in relation to Cortisol AUCg*.
**Supplementary Table 3:** *Model coefficients summary for relationships among Child age, CBCL Internalizing and Child sex in relation to Cortisol AUCg and AUCi*
**Supplementary Table 4:** *Analysis of Variance Table for multilevel model examining relationships among Child age, CBCL Internalizing and Child sex in relation to Cortisol AUCi*.

## Data Availability

Data not available.
