# Supplementary material for: A Cross‐Lagged Panel Analysis of Cortisol Levels and Internalizing Behaviors in Children Born Very Preterm Across Early Childhood: Associations Differ for Boys and Girls at Age 1.5 Years
Source: Dev Psychobiol. 2025 Jul 21;67(4):e70064. doi: 10.1002/dev.70064 (PMC12278196; doi:10.1002/dev.70064)
Supplement: Supplementary file 1 — Supplementary Table 1: Time of cortisol collection at each study visit Supplementary Results Complete reporting of Random Intercept Cross‐lag analyses for Cortisol AUCg, Cortisol AUCi and Internalizing Supplementary Fig.1: Models examining the Bidirectional associations between Cortisol AUCg and Internalizing. (A) Random Intercept CLPM, (B) Random Intercept CLPM model including RI for Internalizing only and (C) CLPM Supplementary Fig.2: Models examining Cortisol AUCg and Internalizing across ages for boys and girls. Bidirectional association Cortisol AUCg and Internalizing for boys and girls, (A) Random Intercept model including Between factor for Cortisol AUCg, (B) Cross‐lag Panel model Supplementary Table 2: Analysis of Variance Table for multilevel model examining relationships among Child age, CBCL Internalizing and Child sex in relation to Cortisol AUCg. Supplementary Table 3: Model coefficients summary for relationships among Child age, CBCL Internalizing and Child sex in relation to Cortisol AUCg and AUCi Supplementary Table 4: Analysis of Variance Table for multilevel model examining relationships among Child age, CBCL Internalizing and Child sex in relation to Cortisol AUCi. [file DEV-67-e70064-s001.docx]

**McLean et al.,** A cross-lagged panel analysis of cortisol levels and internalizing behaviors in children born very preterm across early childhood: Associations differ for boys and girls at age 1.5 years

**Supplementary Materials**

**Supplementary Table 1**

*Time of cortisol collection at each study visit*

|  | **Collection Time (mean +/- SD)** | | |
| --- | --- | --- | --- |
| **Assessment Age** | **Pre-test** | **During** | **End** |
| **1.5 Year CA** | 9:45am (56 mins) | 10:53am (61mins) | 11:24 (58 mins) |
| **3 Year** | 9:13am (38 mins) | 10:53am (59 mins) | 11:25 (62 mins) |
| **4.5 Year** | 9:16am (34 mins) | 11:08am (62 mins) | 11:38 (61 mins) |

**Supplementary Results**

**Cortisol AUCg.** See Supplementary Figure 1. Model (A) fit statistics: χ2 (1) = 1.22, p = .269, CFI = 0.99, RMSEA = 0.03 90%CI [0.00, 2.0], SRMR = 0.02, TLI = 0.97. While an excellent fit, the unstable RMSEA suggests the model may be too closely fitted to the data. Local fit indices indicated good fit (all correlations residuals < .09, with standardized residuals between Cortisol AUCg and Internalizing at 3 years (4.89), and CBCL Internalizing 1.5 and CBCL Internalizing 3 years (-4.12) potentially problematic, however correlations residuals were minimal (.001, -.002 respectively). In Model (B) the Random Intercept of CBCL Internalizing was removed after inspection of model identification indices in Model (A). Model (B) fit statistics indicated good fit, χ2 (3) = 5.03, p = .170, CFI = 0.99, RMSEA = 0.06, 90%CI [0.00, 0.15], SRMR = 0.03, TLI = 0.90, although again the RMSEA confidence interval was wide. Except for the residual correlation between Cortisol AUCg at 1.5 years and Internalizing at 4.5 years of 0.13, all |correlations residuals| <.10 and |standardized residuals| <1.96, suggesting good local model fit. The Cross-lag panel model without Random Intercepts is depicted in Supplementary Figure 2, Model (C). The fit statistics for this model indicated poor fit: χ2 (4) = 10.83, p = .029, CFI = 0.95, RMSEA = 0.01, 90%CI [0.03, 0.17], SRMR = 0.06, TLI = 0.74. Local fit indices indicated poor fit for several relationships (e.g. Cortisol AUCg 1.5 and 3 years, r = .25) and many standardized correlations above 1.96.

The fit of the three nested models was compared using a Chi-Squared Difference Test. While Model (A) (AIC = 4057.9, BIC = 4161.5) and Model (B) (AIC = 4057.7, BIC = 4154.8) indicated no difference in model fit, Δχ²(3) = 3.81, *p* = .149, Model (C) (AIC = AIC = 4061.5, BIC = 4155.4) demonstrated poorer fit to the data compared to Model (A), Δχ²(3) = 9.61, *p* = .022, and Model (B), Δχ²(1) = 9.61, *p* = .016. Similar results were found using the Chi-bar-square difference test of the RI-CLPM (Model (A)) versus the CLPM (Model (C)), Chi-bar-square difference (3) = 9.61, *p* = .011. Model (B) was selected as the model that best fit the data.

**Supplementary Figure 1.**

*Models examining the Bidirectional associations between Cortisol AUCg and Internalizing. (A) Random Intercept CLPMl, (B) Random Intercept CLPM model including RI for Internalizing only and (C) CLPM*


.53

-.41^


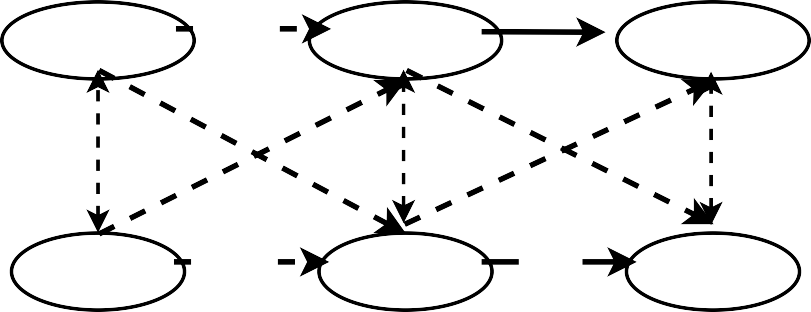


Cortisol AUCg

1.5 Yr

Cortisol AUCg

3 Yr

Cortisol AUCg

4.5 Yr

Internalizing

1.5 Yr

Internalizing .41** 3 Yr

Internalizing

4.5 Yr

Between Cortisol AUCg

Between Internalizing

.40**

(B)

.63***

-.15^

Between Cortisol AUCg

Between Internalizing


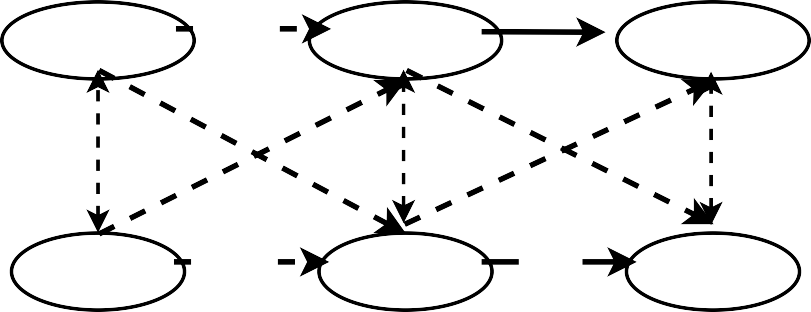


Cortisol AUCg

1.5 Yr

Cortisol AUCg

3 Yr

Cortisol AUCg

4.5 Yr

Internalizing

1.5 Yr

Internalizing .70*** 3 Yr

Internalizing

4.5 Yr

(C)


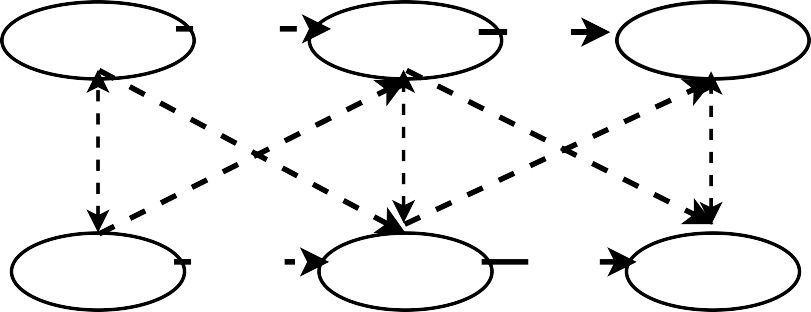


Cortisol AUCg

1.5 Yr

Cortisol AUCg

3 Yr

.37**

Cortisol AUCg

4.5 Yr

Internalizing .59***

1.5 Yr

Internalizing 3 Yr

.60*** Internalizing

4.5 Yr

.59****

Note: Standardized effects (β). All models include gestational age group (ELGA/VLGA) as a covariate. Statistically significant lines are solid, whereas non-significant lines are dotted. *p < 0.05. **p < 0.01. ***p < 0.001.

RI-CLPM examining relationships between Cortisol AUCg and CBCL Internalizing across ages 1.5, 3, and 4.5 years are presented in Supplementary Figure 2. As depicted, for Model (A), the Random Intercept of CBCL Internalizing was unable to be estimated due to negative covariance and therefore was removed. Model (A) fit statistics: χ2 (6) = 5.40, p = .484, CFI = 1.00, RMSEA = 0.00 90%CI [0 – 0.13], SRMR = 0.04, TLI = 1.03 indicating the model had excellent fit indices but was over fitted to the data. All |correlations residuals| <.10 and |standardized residuals| <1.96 for girls and boys, suggesting good local model fit. Examination of data suggested no outliers were driving overfitting. The CLPM was the next simplest model as is depicted in Supplementary Figure 2, Model (B). Model (B) fit statistics: χ2 (8) = 13.31, p = .101, CFI = 0.96, RMSEA = 0.09 90%CI [0.00, 0.16], SRMR = 0.06, TLI = 0.79 indicating reasonable fit. For boys, local fit indices were sound: All |correlations residuals| <.10 and |standardized residuals| <1.96. Local fit for girls was poorer, with high correlations residuals (0.22) between Cortisol AUCg 1.5 and 3 years and Cortisol AUCg 1.5 years and CBCL Internalizing 3 years (0.36), with the latter accompanied by a high standardized residual (1.96). Model (A) (AIC = 4072.8, BIC = 4267.0) demonstrated better fit, Δχ²(2) = 7.92, p = .019, Chi-bar-square diff. (2) = 7.92, *p* = .002 than Model (B) (AIC = 4076.7, BIC = 4264.5). Given Model (A) overfit the data, Model (B) was considered the model with best fit for the data.

# Supplementary Figure 2.

Girls

Girls

*Models examining Cortisol AUCg and Internalizing across ages for boys and girls. Bidirectional association Cortisol AUCg and Internalizing for boys and girls, (A) Random Intercept model including Between factor for Cortisol AUCg, (B) Cross-lag Panel model*

(A) (B)


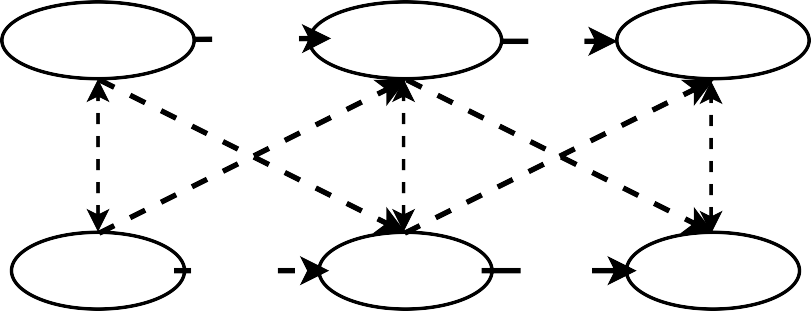


Cortisol AUCg

1.5 Yr

Cortisol AUCg 0.28* 3 Yr

Cortisol AUCg

4.5 Yr

Internalizing

1.5 Yr

Internalizing 0.59*** 3 Yr

Internalizing

4.5 Yr

Boys

Boys


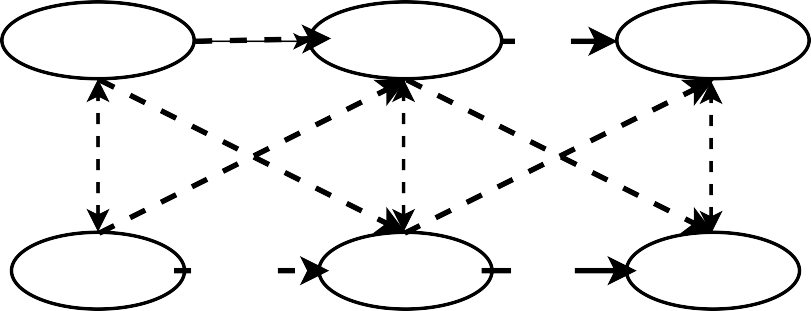


Cortisol AUCg

1.5 Yr

Cortisol AUCg

3 Yr

Cortisol AUCg

4.5 Yr

Internalizing

1.5 Yr

Internalizing 0.60*** 3 Yr

Internalizing

4.5 Yr

Between Cortisol AUCg

.61****


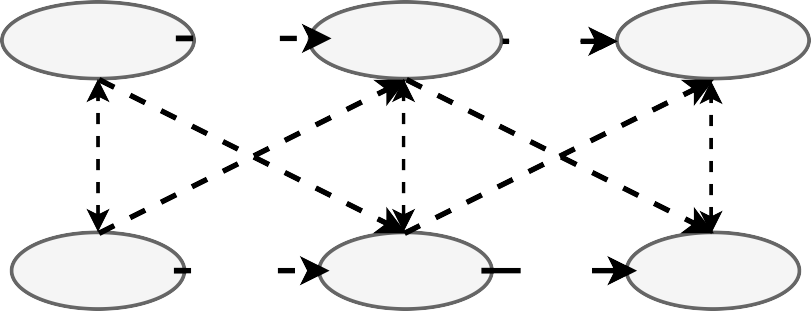


Cortisol AUCg

1.5 Yr

Cortisol AUCg 0.49*** 3 Yr

Cortisol AUCg

4.5 Yr

.30^

Internalizing

1.5 Yr

Internalizing 0.57*** 3 Yr

Internalizing

4.5 Yr


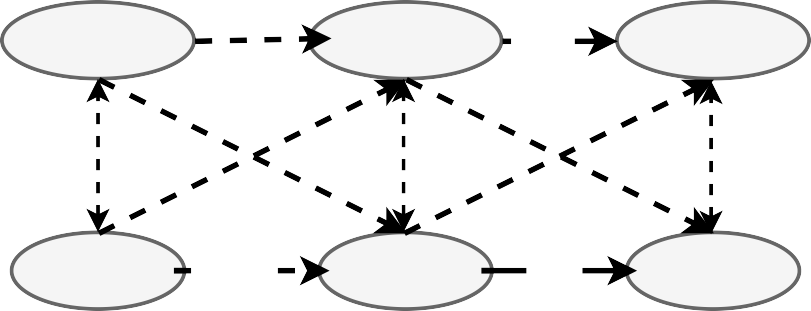


Cortisol AUCg

1.5 Yr

Cortisol AUCg 0.48** 3 Yr

Cortisol AUCg

4.5 Yr

.36^

Internalizing 0.50***

1.5 Yr

Internalizing 3 Yr

0.55***

Internalizing

4.5 Yr

-.77^

-0.35*

.55***

Between Cortisol AUCg

Note: Standardized effects (β). Gestational age group (ELGA/VLGA) included as a covariate. Statistically significant lines are solid. ^p < .10. *p < 0.05. **p < 0.01. ***p < 0.001.

# Cortisol AUCi. Intraclass correlation coefficient (ICC) for Cortisol AUCi was 1.8%, indicating that limited variance in participant AUCi is explained by differences between participants. As such, the RI-CLPM did not include the Random Intercept of AUCi to ensure model estimation. The model demonstrated poor model fit suggestive of overfitting, χ2 (3) = 0.21, p = .977, CFI = 1.00, RMSEA = 0.00 90%CI [0.00, 0.00], SRMR = 0.008, TLI = 1.16. No concurrent or cross-lag relationships were evident. CLPM analysis demonstrated a similarly poor fit to the data. RI-CLPM model fit examining sex differences: χ2 (6) = 2.30, p = .891, CFI = 1.00, RMSEA = 0.00 90% CI [0.00, 0.06], SRMR = 0.025, TLI = 1.22. CLPM model fit examining sex differences: χ2 (8) = 4.88, p = .771, CFI = 1.00, RMSEA = 0.00 90% CI [0.00, 0.08], TLI = 1.14. Both models suggested models were overfitted to the data. No concurrent or cross-lag relationships were evident for boys or girls.

**Supplementary Table 2**

*Analysis of Variance Table for multilevel model examining relationships among Child age, CBCL Internalizing and Child sex in relation to Cortisol AUCg.*

|  | Sum Sq | Mean Sq | NumDF | DenDF | F value | Pr(>F) |
| --- | --- | --- | --- | --- | --- | --- |
| Child Age | 0.05 | 0.03 | 2.00 | 246.10 | 0.12 | 0.889 |
| CBCL Internalizing | 0.14 | 0.14 | 1.00 | 250.90 | 0.66 | 0.417 |
| Child Sex | 0.80 | 0.80 | 1.00 | 231.22 | 3.73 | 0.055 |
| Gestational age group | 1.58 | 1.58 | 1.00 | 115.94 | 7.40 | 0.008 |
| Child age*CBCL Internalizing | 0.05 | 0.02 | 2.00 | 253.53 | 0.11 | 0.897 |
| Child age*Child sex | 1.53 | 0.77 | 2.00 | 246.06 | 3.58 | 0.029 |
| CBCL Internalizing*Child sex | 0.99 | 0.99 | 1.00 | 249.13 | 4.62 | 0.033 |
| Child Age*CBCL Internalizing *Child Sex | 1.57 | 0.79 | 2.00 | 253.50 | 3.68 | 0.027 |

**Supplementary Table 3**

*Analysis of Variance Table for multilevel model examining relationships among Child age, CBCL Internalizing and Child sex in relation to Cortisol AUCi.*

|  | Sum Sq | Mean Sq | NumDF | DenDF | F value | Pr(>F) |
| --- | --- | --- | --- | --- | --- | --- |
| Child Age | 0.01 | 0.00 | 2.00 | 308.57 | 1.27 | 0.282 |
| CBCL Internalizing | 0.01 | 0.01 | 1.00 | 259.14 | 3.35 | 0.069 |
| Child Sex | 0.00 | 0.00 | 1.00 | 240.45 | 0.53 | 0.465 |
| Gestational age group | 0.01 | 0.01 | 1.00 | 129.31 | 5.80 | 0.017 |
| Child age*CBCL Internalizing | 0.01 | 0.00 | 2.00 | 315.85 | 1.46 | 0.233 |
| Child age*Child sex | 0.00 | 0.00 | 2.00 | 308.56 | 0.87 | 0.421 |
| CBCL Internalizing*Child sex | 0.00 | 0.00 | 1.00 | 259.04 | 1.28 | 0.259 |
| Child Age*CBCL Internalizing *Child Sex | 0.00 | 0.00 | 2.00 | 315.82 | 0.83 | 0.435 |

**Supplementary Table 4**

*Model coefficients summary for relationships among Child age, CBCL Internalizing and Child sex in relation to Cortisol AUCg and AUCi*

|  | | |
| --- | --- | --- |
|  | B coefficient (95%CI) | |
|  |  | |
|  | AUCg | AUCi |
|  | | |
| Child Age, 3 years | -1.01^*^ | 0.005 |
|  | (-1.90, -0.13) | (-0.08, 0.09) |
|  |  |  |
| Child Age, 4.5 years | -0.80 | -0.01 |
|  | (-1.68, 0.08) | (-0.09, 0.07) |
|  |  |  |
| CBCL Internalizing | -0.01 | -0.0001 |
|  | (-0.03, 0.002) | (-0.002, 0.001) |
|  |  |  |
| Child sex^a^ | -1.59^**^ | 0.06 |
|  | (-2.64, -0.54) | (-0.03, 0.16) |
|  |  |  |
| Gestational age group^b^ | -0.18^**^ | 0.01^*^ |
|  | (-0.30, -0.05) | (0.002, 0.02) |
|  |  |  |
| Child Age 3 years*CBCL Internalizing | 0.02 | -0.0003 |
|  | (-0.002, 0.04) | (-0.002, 0.001) |
|  |  |  |
| Child Age 4.5 years*CBCL Internalizing | 0.01 | 0.0002 |
|  | (-0.01, 0.03) | (-0.001, 0.002) |
|  |  |  |
| Child Age 3 years*Child Sex | 1.80^**^ | -0.05 |
|  | (0.46, 3.13) | (-0.18, 0.08) |
|  |  |  |
| Child Age 4.5 years*Child Sex | 1.29^*^ | -0.08 |
|  | (0.01, 2.57) | (-0.19, 0.04) |
|  |  |  |
| CBCL Internalizing*ChildSex | 0.04^**^ | -0.002 |
|  | (0.01, 0.06) | (-0.004, 0.0005) |
|  |  |  |
| Child Age 3 years*CBCL Internalizing*Child Sex | -0.04^**^ | 0.001 |
|  | (-0.07, -0.01) | (-0.001, 0.004) |
|  |  |  |
| Child Age 4.5 years*CBCL Internalizing*Child Sex | -0.03^*^ | 0.002 |
|  | (-0.06, -0.003) | (-0.001, 0.004) |
|  |  |  |
| Constant | -1.32^***^ | -0.03 |
|  | (-2.07, -0.57) | (-0.09, 0.04) |
|  |  |  |
|  | | |
| Log Likelihood | -272.34 | 543.01 |
| Akaike Inf. Crit. | 574.68 | -1,056.03 |
| Bayesian Inf. Crit. | 631.62 | -997.04 |
| *Note:* ^a^ Child sex, boy = 0, ^b^Extremely low gestational age = 0 | ^*^p<0.05; ^**^p<0.01; ^***^p<0.001 | |
